# Specialty Grand Challenge – Pediatric Pulmonology

**DOI:** 10.3389/fped.2013.00014

**Published:** 2013-07-01

**Authors:** Anne B. Chang

**Affiliations:** ^1^Queensland Children's Respiratory Centre, Royal Children's Hospital, Queensland Children's Medical Research Institute, Queensland University of TechnologyBrisbane, QLD, Australia; ^2^Child Health Division, Menzies School of Health ResearchDarwin, NT, Australia

In William Silverman's book, “Where's the evidence” (1), he elegantly espouses the need for, and the contributions of, good research to improve clinical outcomes. But with respect to technological advancements, he challenges us and future generations on where the line between “knowing (the acquisition of new knowledge) and ‘doing’ (the application of the new knowledge)” should be drawn (1). Just because we can do things, does not mean that it should be done without considering the moral and social consequences.

On a world-wide scale, the respiratory system is a particularly important field in childhood. Key reasons include; (i) pneumonia remains the biggest killer of children, despite massive advances in medicine, vaccinations, and public health; (ii) unlike some other organs, the lung continues to grow at least till 7–8 years of age, if not longer; and (iii) pulmonary immunity and respiratory phenotype is influenced by genetic-environmental interaction that commence very early in life (possibly *in utero*). Thus, it is highly biologically plausible that many common conditions in adulthood emerge from early childhood factors (2). There is indeed increasing evidence that a substantial proportion of lung disease in adults (such as chronic obstructive lung disease and bronchiectasis) has its roots in childhood (where it is potentially reversible) (3, 4). To reduce the world-wide burden of chronic respiratory illness, a greater focus on children's lung and generic health is required. Many, if not most of these diseases, are potentially modifiable through clinically based interventions or are preventable.

Yet, there is comparatively little research for the huge word-wide burden of respiratory illnesses. This is compounded by the fact that the socio-economic gradient is a factor in many diseases of the respiratory system, i.e., people in resource-poor countries and the underprivileged in resource-rich countries have the highest burden of respiratory illness. While this represents a challenge it also presents an opportunity for those in resource-rich countries to find interventions that can be applied to resource-poor settings.

Indeed, while major advances in child health are likely to follow improvements in education and reductions in poverty, a systematic overview of interventions addressing the social determinants of health found a striking lack of reliable evaluations (5). Where evidence was available, the health improvement associated with interventions was modest or uncertain (5). Thus advances in health care for important childhood conditions that are achievable within a much shorter timeframe remain essential. Interventions that can be feasibly implemented remain vital to improve world-wide lung health. Also, while the greater world-wide challenge is to improve lung health in developing countries, respiratory illness remains the most common cause of hospitalization in children in affluent countries. This highlights the fact that while it remains imperative that to tackle the social determinants of health, it is insufficient to just focus on it. Nevertheless, equity of service is important. Even in affluent countries, equity of high quality service for different diseases [such as comparing service for people with cystic fibrosis (CF) with that for those with non-CF bronchiectasis] and different settings (e.g., urban vs. remote settings) remains problematic and a challenge.

While there is little doubt that solutions to improving lung health will need to include technological advancements, in some settings substantial improvements may be possible with less expensive programs. This could occur through challenging existing paradigms. The major improvement in longevity of people with CF over the last three decades is not related to major technological or genetic breakthroughs, but related to improved clinical care through better attention to nutrition, airway clearance and intensive treatment of infections. By changing the paradigm of a defeatist approach of “minimal treatment” to a “best possible care of intensive treatment,” a reduction in early morbidity and mortality have occurred in CF in affluent countries and non-CF bronchiectasis in Indigenous populations in affluent countries.

Researchers will constantly be reminded that despite the massive investment in research in asthma pathophysiology, the largest contributor to improvements in asthma morbidity and mortality in affluent countries was patient-centered education and better use of available current medications (6, 7). It is indeed ironical that while many renowned researchers discovered and paddled the importance of allergy and its role in the pathophysiology in the field of asthma over decades, the prevalence of asthma and its severity in affluent countries was actually declining. This highlights the need for a multi-disciplinary collaborative approach to make a real impact, as well as the importance of a translational approach in research.

Research in other areas such as the diagnosis and treatment of pneumonia and its consequences are required. While it may appear simple with the use of chest radiography and antibiotics, issues of how best to diagnose and treat so as to prevent future chronic lung disease such as COPD (8) and bronchiectasis (9), especially in resource-poor settings, remains a great challenge. While COPD is related with tobacco smoke exposure in some people, a large proportion of COPD is unrelated to tobacco smoke.

Studies on how best to prevent lung diseases, above and beyond tobacco control is one possible key in improving future lung health of children. Factors that govern lung development and growth, pulmonary innate and adaptive immunity and their relationship with the lung microbiome, metagenomics, and metabolomics (etc) will likely contribute to future clinical interventions and hence lung health.

Recent studies have shown that some respiratory disease and infection have unique airway and/or systemic molecular signatures. These gene expression signatures may be a powerful tool allowing earlier detection of certain diseases at their earliest stages before overt clinical manifestation when they are most preventable. Molecular signatures and biomarkers represent the future of many fields of medicine, including respiratory medicine where disease diagnosis, treatment, monitoring, and prevention may be guided by a patient's unique composition and/or response. However, all advances need to be balanced with evidence that is not driven by those interested only in financial procurement.

“Frontiers in Pediatric Pulmonology” will stimulate and support researchers involved in the most recent advances in the field with a translational application. The grand challenge for clinicians and researchers in years to come include:

- Discovering affordable technological advances for point of care testing of common illness such as pneumonia that allows targeting of better use of antibiotics;
- How to interest researchers to undertake studies and granting bodies to fund studies on common conditions such as acute cough and pneumonia?
- How best to support behavior changes that lead to improving adherence to effective evidence-based interventions such as the use of inhaled corticosteroids for chronic asthma and hygiene for reducing acute respiratory infections?
- What are the best and long lasting interventions to prevent the uptake of tobacco smoke in the young?
- Can new drug discoveries that are effective and safe, be more equitably available?
- Can and how will the blooming world of “omics” contribute to the betterment of lung health?
- Can safe and feasible interventions to reverse lung disease in childhood (such as bronchopulmonary dysplasia) be found?
- Will current research on the fetal treatment of pulmonary defects prevent future congenital lung anomalies?
- Can we expect the combination of experimental studies, computational models and nanotechnologies to unveil the remaining secrets of lung and function?
- Will robust gene expression signature or biomarkers to diagnose and/or monitor the various lung diseases be discovered?
- Which mechanisms and interventions are most important in optimizing lung growth and development?
- Can clinicians, public health experts, the pharmaceutical industry, and political masters join forces to abolish the barriers to improving global lung health?
- Will the dream of equal high quality and holistic care irrespective of setting and disease become a reality?

All researchers and scientists interested in contributing to improving lung health especially that affecting the most disadvantaged in the world will find that an open platform for mutual exchanges beneficial. One of the aims of “Frontiers in Pediatric Pulmonology” is to fulfill this role and attract the most visionary people to discover and share unexpected insights, breakthroughs and advance this field for the betterment of humanity. The coming years will give us the opportunity foster scientific and affordable technological innovations to solve respiratory problems in children which will also reduce adult lung disease. In doing so, it would also impact on future cardiovascular health as chronic wet/productive cough is a known independent risk factor of cardiovascular disease (10).
